# App review driven collaborative bug finding

**DOI:** 10.1007/s10664-024-10489-x

**Published:** 2024-07-26

**Authors:** Xunzhu Tang, Haoye Tian, Pingfan Kong, Saad Ezzini, Kui Liu, Xin Xia, Jacques Klein, Tegawendé F. Bissyandé

**Affiliations:** 1 SnT, University of Luxembourg, Luxembourg City, Luxembourg; 2 Huawei, Hangzhou City, China; 3 School of Computing and Communications, Lancaster University, Lancaster, UK

**Keywords:** Bug finding, App review, Bug similarity, Bug report

## Abstract

Software development teams generally welcome any effort to expose bugs in their code base. In this work, we build on the hypothesis that mobile apps from the same category (e.g., two web browser apps) may be affected by similar bugs in their evolution process. It is therefore possible to transfer the experience of one historical app to quickly find bugs in its new counterparts. This has been referred to as collaborative bug finding in the literature. Our novelty is that we guide the bug finding process by considering that existing bugs have been hinted within app reviews. Concretely, we design the BugRMSys approach to recommend bug reports for a target app by matching historical bug reports from apps in the same category with user app reviews of the target app. We experimentally show that this approach enables us to quickly expose and report dozens of bugs for targeted apps such as Brave (web browser app). BugRMSys ’s implementation relies on DistilBERT to produce natural language text embeddings. Our pipeline considers similarities between bug reports and app reviews to identify relevant bugs. We then focus on the app review as well as potential reproduction steps in the historical bug report (from a same-category app) to reproduce the bugs. Overall, after applying BugRMSys to six popular apps, we were able to identify, reproduce and report 20 new bugs: among these, 9 reports have been already triaged, 6 were confirmed, and 4 have been fixed by official development teams.

## Introduction

Modern apps must evolve quickly to adapt to a fierce competition in app markets where users have varied choices among feature-rich apps (McIlroy et al. 2016). Unfortunately, the fast iteration in app updates often results in defects being found by users after releases (Calcagno et al. 2015). Various research efforts based on static analysis (Jiang et al. 2017; Lee et al. 2016; Talukder et al. 2019) and dynamic testing (Hu et al. 2014; Van Der Veen et al. 2013; Fan et al. 2018; Su et al. 2020; Liu et al. 2022) have therefore been carried out to detect bugs before releasing apps. Bug-free apps remain however a myth and even popular apps, which are intensively used by large user communities, often display simple but annoying defects (Fan et al. 2018; Amalfitano et al. 2018; Sun et al. 2021). Through app reviews, users can provide feedback on buggy behaviour that sometimes go overlooked by app developers for various reasons: reviews can be redundant and uninformative (e.g. simple praise or dispraise repeating the star rating) (Maalej et al. 2016). App reviews are also time-consuming to exploit and can mislead the identification of fault locations (Stanik et al. 2019). In contrast, official bug reports filed in the issue tracker are the focus of developer communities since these reports tend to be more readily exploitable for bug resolution.

It is noteworthy that if recurring bugs are not swiftly addressed by developers, they will lead to negative app reviews with significant impact on app score in app markets and other severe consequences such as app fails (Li et al. 2010). The aforementioned situation calls for a more careful consideration of user reviews by developers. In particular, it would be appealing to translate app reviews into bug reports that can be used by developers as starting points in their fight against bugs. However, there exists a significant gap between the language of user reviews and the language of developer bug reports. The former is generally formal and technically-written while the latter is informal and colloquially-written. In a recent work,  Haering et al. (2021) proposed a deep learning approach to match app reviews and bug reports with the ambition of easily tracking whether an issue reported in app review was already filed as an official bug report, which should increase bug fixing priority. While we subscribe to the claim that user feedback often lacks information that is relevant for developers (such as steps to reproduce or affected versions) (Martens and Maalej 2019; Zimmermann et al. 2010), their approach (1) does not address the key problem of review deluge, and (2) misses the opportunity to reveal new bugs to the developers. Indeed, on the one hand, for a popular app, there can be thousands of new reviews every day, most of which are noisy for developers since they do not offer insights into bugs. On the other hand, some app reviews may actually mention important and annoying bugs which can impact user experience for a large number of users without ever being reported formally in the issue tracker.

In another research direction,  Tan and Li (2020) have proposed Bugine (Tan and Li 2020), a collaborative bug recommendation system that aims at pairing similar issue reports across different apps. Thanks to Bugine, they have empirically shown that it is indeed possible to match similar issue reports across different apps. However, Bugine can only report issues across apps where the relevant UI design is of high visual similarity. Besides limited to only UI-related bugs, Bugine does not take target app’s review into consideration, which enable it no ability to pick up useful bugs as input.

Building on the hypothesis proposed by Tan et al., we performed a preliminary study (cf. Section 2) to investigate whether apps within the same category (e.g., two web browser apps or two calendar apps) tend to encounter similar development issues. Indeed, our observations suggested that apps in the same category share common challenges since these apps (1) are developed using similar development frameworks for comparable functionalities (e.g., *Unity* for gaming apps), (2) employ similar UI design logic, and (3) use the same storage/notification/hosting services (e.g., FireBase) (Long et al. 2014, 2016). This leads us to believe that learning from the experiences of existing apps could be promising for improving new ones.

Drawing upon this observation, we delved deeper into the potential of shared knowledge across apps within the same category. Prior work (Li et al. 2019; Bevan et al. 2002) indicates that interactions among developers of different software can effectively enhance the quality of each piece of software. We conceive of these interactions in our context as the sharing of issues and resolutions among apps within the same category. These shared experiences, articulated in the form of bug reports, constitute a valuable resource for continuous learning and improvement for each app within the category. This understanding serves as the foundation for our unique strategy of collaborative bug-finding, which is driven by user app reviews.

**Fig. 1:**
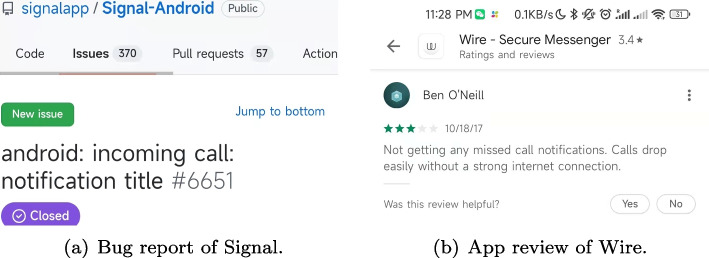
Example of bug report and app review matched by BugRMSys

**This paper** We hypothesize that if app **A** and app **B** belong to the same category (we consider the categories listed in the Wikipedia enumeration of popular free and open source Android apps^1^, e.g., web browsers, Games, etc.), bug reports from one can be relevant for discovering bugs in the other. Unfortunately, there can be too many bug reports filed in some categories of applications. For example, in the Web Browser category, the Firefox issue tracker alone has received more than 20,000 bug reports. It is, therefore, necessary to identify those issues that are more likely to be relevant for the app under study (i.e., the target app for bug discovery). To that end, our novel strategy in this work is to explore app reviews written by users for the target app. Our idea is that app reviews, which may contain hints about buggy behaviour observed by users of the target app, can be matched to bug reports from other apps in the same category.

**Fig. 2:**
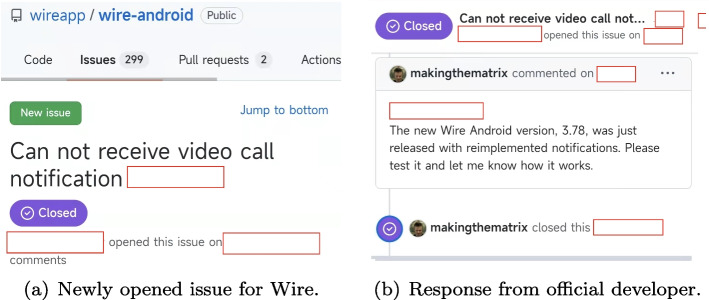
Bug found by BugRMSys and response from official developer (some texts have been hidden for privacy protection)

We propose BugRMSys, a collaborative bug-finding approach that is guided by user app reviews. BugRMSys finds bugs by recommending a bug report of app **A** (e.g., the excerpted bug report of Signal in Fig. 1(a)) as being relevant to the target app **B** (i.e., Wire) given the similarity of the bug report with app reviews from users of **B** (e.g., the excerpted user review of Wire in Fig. 1(b)). With the app review in **B** matched with a similar bug report in **A**, we reproduce the bug in **B** by leveraging reproduction steps in the bug report and additional information details from the app review. If reproduction is successful, we can confirm having found a “new bug” that will be filed into the official issue tracker for app **B**. For example, the corresponding bug found by BugRMSys in Wire was reported to its developers (shown in Fig. 2(a)), and was finally got fixed by Wire’s official developer (Fig. 2(b)) in one day. Surprisingly, the relevant user app review had been submitted since four years, but there is no any corresponding bug reported in the official issue tracker of the app yet. To the best of knowledge, this scheme of collaborative bug finding driven by app reviews, has not been previously explored in the literature.

Our approach represents a novel contribution to the field as it adopts a unique collaborative method, utilizing data from same-category apps. This approach not only leverages the commonalities within the same app category but also allows for an unprecedented comparison and bug identification process. This hasn’t been done before and provides a new perspective in app bug detection and recommendation.

The main contributions of our work are as follows:

- We present insights from an empirical study about the similarity of issues reported across apps from the same category. These insights provide the intuitive basis for collaborative bug finding.
- We devise BugRMSys , a bug recommendation system, which leverages similarities of user app reviews with bug reports to identify which bug reports from same category apps are good candidates to attempt a bug reproduction on a target app.
- We demonstrate experimentally the effectiveness and usefulness of BugRMSys : applied to 6 apps from different categories, we find and reproduce 20 new bugs (i.e., bugs that are not yet reported in the issue trackers of these apps): 9, 6, and 4 reports have been already triaged, confirmed, fixed by official development teams, respectively.
- Through a rigorous evaluation involving independent experts, we demonstrated a high relevance between bug reports identified.

## Preliminary Study

In this section, we conduct a preliminary study to evaluate the hypothesis of our work. Specifically, we seek to check that apps from the same category are more likely to share similar bug reports (and thus a bug report from app A could be relevant for app B if A and B are from the same category). We focus the comparison by estimating the overlap (i.e., the proportion of common words) between bug reports. To that end, we analyze the overlap rate of top$_K$ frequent words of reports for apps from same or different categories. From a more qualitative perspective, we also analyze which types of words are frequently shared.

### Empirical Setup

**Apps: ** To conduct our preliminary study, we consider six popular apps listed in the Wikipedia page of free and open-source Android apps. Table 1 summarizes statistics about these apps. We consider *Signal* and *Wire* within the *Privacy-Security* category. Both apps have been downloaded more than one million times from the Google Play store. We also consider *Firefox* and *Brave*, two widely popular in the *Web Browser*, category. Finally, we consider *Nextcloud* and *Owncloud* among the apps in the *Office Suite* category.

**Table 1:** The Apps and their categories

App Name	Repo Name	Category	# Downloads	# Bug Reports
Signal	Signal-Android	privacy security	>50 million	11,980
Wire	wire-android	privacy security	>1 million	3,677
Firefox Android	fenix	Web Browser	>100 million	24,087
Brave	brave-browser	Web Browser	>50 million	21,436
NextCloud	nextcloud	Office Suite	>1 million	9,890
owncloud	owncloud	Office Suite	>0.1 million	3,571

**Bug Reports: ** For each app, we have collected all bug reports that are present in their issue tracker system. The number of collected bug reports is given in the last column of Table 1. Note that, for our experiment, we employ the Python NLTK (Loper and Bird 2002) library and self-defined filters to pre-process (This is detailed in Sec A.5) the bug reports for natural language processing. We apply typical pre-processing tasks to remove stop words (Wilbur and Sirotkin 1992), punctuation, digits, etc. (Haddi et al. 2013). Meanwhile, to limit experimental bias, we set 10 different sizes *K* for the set of most frequent words, increasing step-wise until an order of magnitude: we consider Top$_{100}$, Top$_{200}$,..., Top$_{1000}$ frequent words. Concretely, to build each Top$_K$ set for each app, we extract the *K* most frequent words in its bug reports. By analysing the Top$_K$ frequent words, we can assess the differences in shared words between bug reports of same-category apps and different-category apps. Applied to all set ten Top$_K$ sets, we can further check for potential trends, while empirically identifying the value of *K* under which the overlap (i.e., the proportion of shared words) is the highest.

**Notation: ** In the rest of this paper, the category Privacy-Security, the category Web Browser, and the category Office-Suite are referred to as PS, WB, and OS, respectively. We also note the three pairwise combination of apps from the same category as follows: PS-PS: <Signal, Wire>; WB-WB: <Firefox, Brave>; OS-OS: <Nexcloud, Owncloud>. Similarly, we consider 12 pairwise combinations of apps from different categories as follows: PS-WB: <Signal, Firefox>, <Signal, Brave>, <Wire, Firefox>, and <Wire, Brave>; PS-OS: <Signal, Nextcloud>, <Signal, Owncloud>, <Wire, Nextcloud>, and <Wire, Owncloud>; WB-OS: <Firefox, Nextcloud>, <Firefox, Owncloud>, <Brave, Nextcloud>, and <Firefox, Owncloud>.

**Overlap Rate (Metric):** Given two sets *X* and *Y*, Overlap rate of *X* with *Y* is computed as follows:

1 $$\begin{aligned} Overlap(X_Y) = \frac{size(\left| X \cap Y \right| )}{size(X)}, \end{aligned}$$

where *size* denotes the size function for sets. If both sets have the same size (e.g., in our case, we select top$_K$ frequent words in the different sets of bug reports), then $Overlap(X_Y)$ $=$ $=$ $Overlap(Y_X)$.

### Hypothesis Analysis

In this section, we check whether our hypothesis is valid from the quantitative and qualitative aspects of bug reports.

**Fig. 3:**
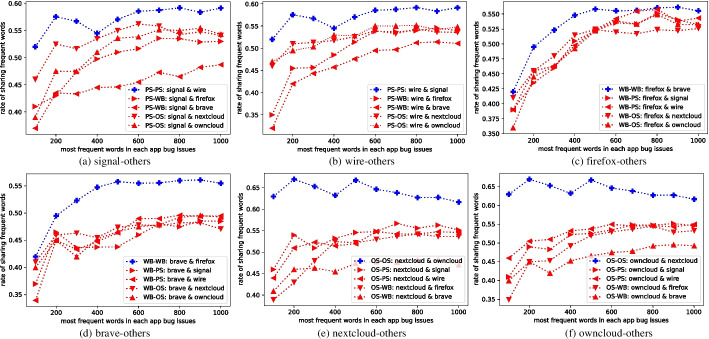
Overlap rate of hot words of category apps’ bug issues

**Fig. 4:**
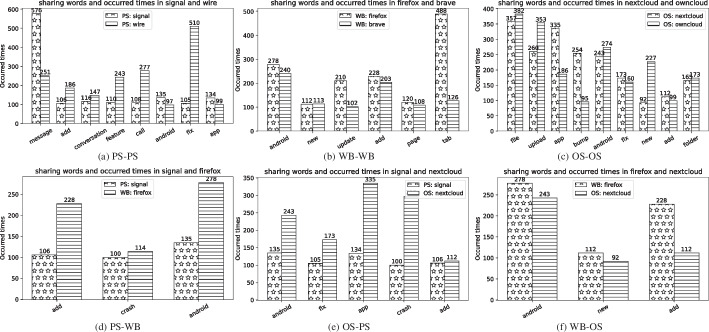
Distribution of Top$_{20}$ shared frequent words in same-category app pairs and different-category app pairs

**Quantitative Analysis:** The results of the quantitative study are presented in Fig. 3, where for each app X, we compute the percentage of overlap, i.e., the percentage of shared words between the Top$_K$ frequent words in the bug reports of X and the Top$_K$ frequent words in the bug reports of another app Y. Our analysis reveals that the percentage of shared Top$_K$ frequent words is highest when apps X and Y are from the same category. We further delved into the nature of these shared words, differentiating between general/common words and specific, bug-related terms, to understand their role in the context of bug reports (Fig. 4).

**Fig. 5:**
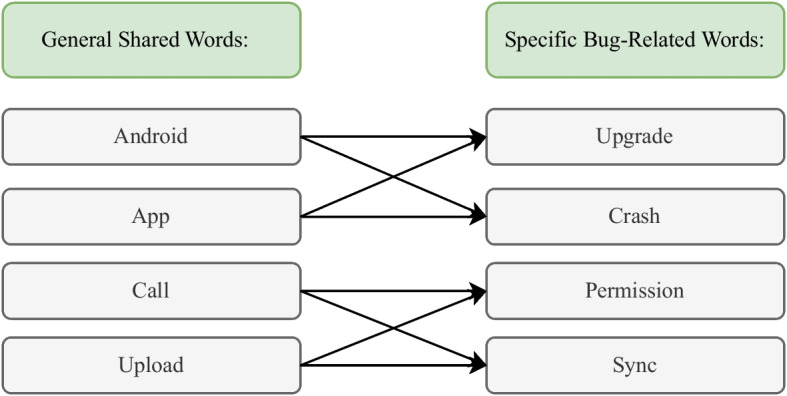
Examples indicating the relationship between general shared words and specific bug-related words

**Qualitative Analysis:** In the qualitative analysis, we set a threshold of frequency as 20 to select the most frequent words in bug reports. With the Top$_{20}$ most frequent words of each app, we assessed to what extent different app pairs share these words, and how often the shared words occur in each app. A significant observation is that while many shared words are general (e.g., ‘android’, ‘app’), their contextual use within bug reports can still provide valuable insights. We examined the context in which these general words appear, exploring how they relate to more specific bug-related terms. This analysis helped us understand how general terms contribute to identifying patterns or categories of bugs within the same app category. As shown in Fig. 5,we take examples to explain the relationship between general shared words and specific bug-related words: Normally, each general shared word can be matched to multiple specific bug-related words to help confirm where the bug is happening. For example, ‘Upgrade’ and ‘Crash’ could indicate the problem in ‘Android’ or ‘App’, but they can not mean the problems of ‘Call’. ‘Permission’ and ‘Sync’ could refer to the issues of ‘Call’ or ‘Upload’, but not possible refer to the more general problem in ‘Android’ or ‘App’. Here, for example, the title of the bug issue: “it crashes frequently, since upgrading my Android". In this report, “Android" sets the context to the Android platform, indicating that the issue is related to or observed on Android devices. The term “Upgrade” further localizes the problem to a specific action or event — the upgrading of the Android OS. This combination of terms suggests that the problem is not just related to the app’s performance in general but is specifically triggered or exacerbated by upgrading the Android OS (Table 2).

To make this observation more clear, we provide examples as shown in Fig. 5.

**Table 2:** Top$_{20}$ frequent words in bug reports for each app

App		Top$_{20}$ frequent words
PS	Signal	signal, message, sms, group, android, app, contact, send, mms, conversation, notification, feature, call, add, fix, request, crash, textsecure, text
	Wire	fix, feature, add, conversation, avs, part, message, bump, wire, new, update, call, user, remove, app, android, video, version
WB	Firefox	bug, fnx, android, tab, add, tabs, update, search, issue, menu, components, fenix, button, page, crash, new, ui, strings, version
	Brave	brave, x, release, android, chromium, add, desktop, test, run, rewards, manual, ads, tab, browser, upgrade, wallet, new, settings, page, update
OS	Nextcloud	app, upload, bump, android, crash, file, nextcloud, fix, folder, auto, stable, add, error, rc, new, use, account, server
	Owncloud	android, upload, new, file, app, folder, fix, owncloud, feature, release, bug, request, update, share, add, view, arch, bump

We note that the non-shared frequent words of the same-category app pairs often relate to general features or problems, whereas those in different-category pairs are more aligned with their main features. For instance, in the pair <Signal, Firefox>, the non-shared words in Firefox (e.g., ’tabs’, ’browser’) are associated with traditional features of web browsers.

To sum up, the shared information in the bug reports of same-category apps not only indicates a higher degree of similarity but also shows how general words, when analyzed in the right context, can contribute significantly to bug categorization and identification.

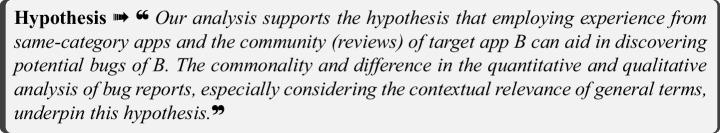



**Fig. 6:**
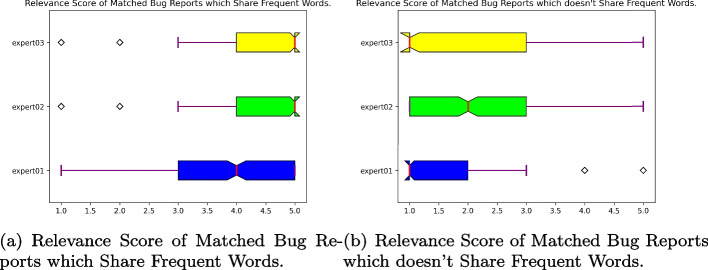
If matched bug reports are highly similar, the relevance score will be 5, if the matched bug reports are no related, the relevance score will be 1

### Expert Analysis on Hypothesis

In our study, we engaged three independent experts to manually score the relevance of matched reviews and bug reports. These reports were formed into two distinct groups. The first group comprised pairs of reports that share frequent words, and the second group comprised pairs that share few to no common words. Each expert evaluated a sample of 385 pairs from each group, drawn randomly from a population of one million pairs. This sample size was calculated to provide a 95% confidence level and a 5% margin of error.

The scores assigned by the experts ranged from 1 to 5, with a score of 1 indicating no relation between the bug reports, and a score of 5 indicating a high relevance. For the first group, the experts’ mean scores were approximately 3.85, 4.23, and 4.47 for Experts 1, 2, and 3, respectively. This suggests a high degree of relevance for bug reports that share frequent words. The boxplot in Fig. 6(a) provides a visual representation of these scores.

In contrast, for the second group, the experts’ mean scores were significantly lower, with values of approximately 1.65, 2.01, and 1.87 for Experts 1, 2, and 3, respectively. This indicates a low degree of relevance for bug reports that do not share frequent words, as shown in Fig. 6(b).

**Fig. 7:**
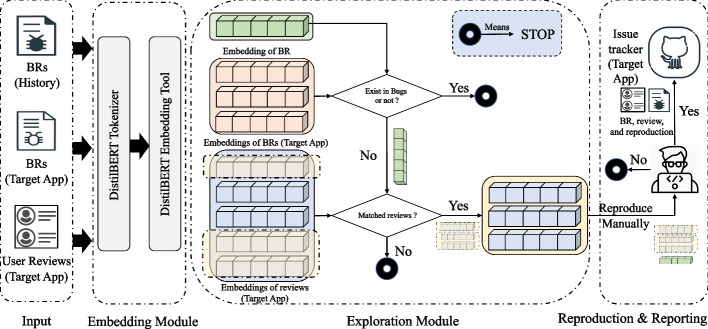
Overview of BugRMSys

These results were further adjusted to account for individual bias, resulting in final mean scores of 1.65, 2.01, and 1.87 for Experts 1, 2, and 3, respectively. This rigorous manual evaluation by independent experts strengthens the validity of our study and provides valuable insights into the relationship between bug reports and reviews (Fig. 7).

## BugRMSys

**Fig. 8:**
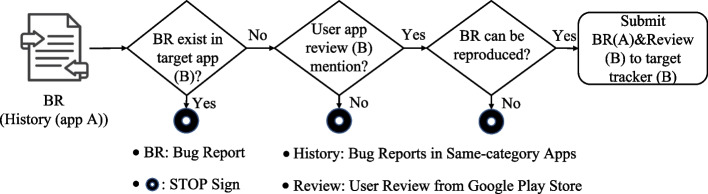
General workflow of app review driven collaborative bug finding

Figure 8 depicts the general workflow of the approach for app review driven collaborative bug finding.

### Notation

we will refer to app A and app B as two apps that belong to the same category.

Our approach attempts to leverage the development experience of the historical app A to find bugs in the target app B. The workflow therefore starts with a representative bug report that has been handled in the development of app A. If a similar bug report exists in the issue tracker of the target app B under study, the bug finding process is halted and must be restarted with another bug report from app A. Otherwise, the workflow proceeds to check whether the bug report content is similar to some user reviews (B) (that has therefore gone overlooked). If one or several relevant app reviews are found, we must attempt to reproduce the bug in app B based on reproduction steps in the bug report of app A as well as specific details in user review of B. In our evaluation, once the buggy behavior is confirmed through reproduction of the bug report, we further submit a new bug report in the issue tracker of app B. In the remainder of this section, we will present a real-world example before detailing the technical approach for bug recommendation.

### Running Example

**Fig. 9:**
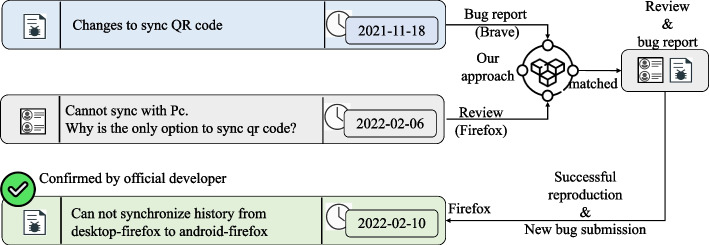
Running example of BugRMSys

Figure 9 illustrates the case where we leveraged BugRMSys to discover a new bug in the web browser app Firefox. By iterating over bug reports from the active development repository of Brave, we identified a bug report which refers to synchronization with QR Code. A similar bug report was absent from the issue tracker of Firefox. After matching by BugRMSys , a user review of Firefox had clearly stated a similar problem “*Cannot sync with Pc. Why is the only option to sync qr code?*”. With the user’s assessment, the bug report might indeed be relevant, we thus explore the steps enumerated in Brave’s bug report to reproduce the matched problem in Firefox. Surprisingly, the bug was successfully reproduced within a few minutes. We then submitted a bug report with two screenshots into the issue tracker of Firefox, which was eventually confirmed by the Firefox development team in 4 days.

Intuitively, our bug recommendation could have started with considering available user app reviews and try to correlate with historical bug reports from other apps in the same category of the target app. Unfortunately, in practice, most app reviews do not provide usable information. Therefore, we propose to initiate the search with the bug reports, which are in lesser numbers, and are more structured. Nevertheless, many bug reports in the same category as the target app are actually raising irrelevant issues. Therefore, it is important to further check if such potential issues have caught user attention and lead them to write reviews that mention them. This motivates the need to devise a reliable mechanism to precisely match useful app reviews with relevant bug reports.

### Approach

Figure 7 illustrates the details of automatically finding bugs with BugRMSys , which includes two main modules: an embedding module and an exploration module. The remainder of this section describes how the embedding module deals with bug report and app review text representation, and how the exploration module eventually identifies relevant bug reports for recommendation.

#### Embedding and Processing Techniques

To embed app reviews and bug reports into vector representations for semantic similarity computation, we use state-of-the-art pre-trained deep learning models, specifically DistilBERT (Sanh et al. 2019), a lighter but efficient version of BERT (Devlin et al. 2018). This choice addresses several challenges:

1. App reviews and bug reports differ in organization and vocabulary (technical vs. non-technical terms). Reviews often contain errors, emoticons, non-technical references, and can be repetitive. Bug reports follow a technical template with detailed descriptions, including technical terms and are often lengthy, making it challenging to capture overall semantics.
2. To mitigate these issues, for app reviews, we apply NLP pre-processing techniques such as lowercasing tokens, removing punctuation, stop words, digits, and emoticons. Reviews shorter than 10 words are dropped, as they typically lack substantial content. For bug reports, we focus on the titles, which encapsulate essential content, avoiding the noise from detailed descriptions.

DistilBERT is selected for its performance and practicality. It requires fewer resources and less training time than BERT while maintaining similar capabilities. It uses a byte pair encoding method for tokenization, effectively handling “out of vocabulary” issues and recognizing compound words. The DistilBERT model produces a 768-dimensional contextualized embedding for each token, considering the surrounding words’ context. This approach is crucial for accurately representing the content of app reviews and bug reports.

The embedding process involves tokenization using DistilBERT’s WordPiece tokenizer, conversion to input IDs, embedding lookup, and positional encoding. The model’s architecture includes Transformer blocks with self-attention mechanisms, enabling it to capture complex semantic relationships within texts. The final embeddings from DistilBERT are used for calculating document cosine similarity, facilitating the semantic comparison of app reviews and bug reports.

#### Exploration

Once the embedding module has produced numeric representations for bug reports and app reviews, the exploration module attempts to identify those reviews that are relevant for a given bug report. If a user review is found, the bug report can be recommended (supposing that no similar bug report was already filed for the target app).

To this end, following empirical findings from previous studies (Ghosh and Strehl 2006) dealing with text similarity metrics (Wooditch et al. 2021), we resort to employing the cosine similarity (Sitikhu et al. 2019) between low-dimensional representations of bug reports and app reviews to measure their relatedness. BugRMSys then prioritizes app reviews as being potentially relevant or not by referencing a threshold value to dismiss app reviews that should not be listed. Therefore, if one app review of B is found to be similar with a bug report in A, the bug report will be recommended as describing a bug that is relevant for the target app B. Otherwise, the bug report will not be recommended. The threshold setting is presented in Section 5.1.

## Experimental Design

### Research Questions

Our experiments aim at answering the following research questions:

- **RQ-1: To what extent is the** BugRMSys **bug recommendation approach driven by app reviews effective?** Our approach is devised to recommend bugs with our hypothesis. In this RQ, we first investigate to what extent app reviews can be used to recommend the bug report for the target app. Before that, we build a “ground truth” set of bug reports pairwise combinations (from a target app and a same category app).
- **RQ-2:** **Can** BugRMSys **expose bugs in real-world apps based on existing app reviews?** With this research question, we explore the feasibility of exposing bugs in real-world apps by BugRMSys with 20 apps of 9 categories. We investigate both the Exploration module for bug recommendations as well as the actual bugs that developers can confirm.
- **RQ-3:** **How does** BugRMSys **compare against previous bug recommendation systems?** With this research question, we compare the bug recommendation approach of BugRMSys against the bug recommendation systems (DeepMatcher (Haering et al. 2021) and Bugine (Tan and Li 2020)) in three dimensions (inputs, automation, effectiveness).

### Dataset

We investigate the feasibility of our hypothesis with BugRMSys , we first curate a dataset that collects bug reports and app reviews from 20 free and open-source apps of 9 categories. For each app, we collect bug reports from its GitHub issue tracking system. App reviews are selected by checking whether an app review is related to a concrete bug report. App reviews are further curated following the order given after a sort on “helpful”. A review is given a “helpful” score according to the number of users who agree with it. In a recent study, Häring et al. (Haering et al. 2021) have used this score to measure the importance of a review. Note that, collected bug reports and app reviews are written in English. Table 3 summarizes the information of collected data.

**Table 3:** Dataset Summary

App Category	# Apps	App Names	Size	Popularity (#download)	# Bug Reports (all)	# App Reviews (all)
privacy security	2	Signal	56Mb	100M$+$	15,657	15,428
		Wire	100Mb	1M$+$		
web browser	2	Firefox	86Mb	100M$+$	45,523	166,360
		Brave	225Mb	100M$+$		
office suites	2	Owncloud	25Mb	100K$+$	13461	2,260
		Nextcloud	32Mb	1M$+$		
emulator	3	Dolphin	18Mb	5M$+$	25,822	43,709
		Mupen64plus	15Mb	10K$+$		
		Ppsspp	32Mb	100M$+$		
communication	2	Jitsi	53Mb	10M$+$	4,555	3,166
		ConnectBot	3Mb	1M$+$		
game	2	Pxiel	37Mb	1M$+$	1,590	3,098
		Mindustry	62Mb	5M$+$		
multimedia	2	AntennaPod	9Mb	500K$+$	5,898	53,746
		NewPipe	9Mb	50K$+$		
reading	2	Kiwix	19Mb	1M$+$	3,704	62,755
		FBReader	9Mb	10M$+$		
science and education	3	Sky Map	44Mb	50M$+$	16,045	36,413
		Stellarium	5Mb	10M$+$		
		AnkiDroid	83Mb	10M$+$		
Total	20	-	-	-	132,255	386,935

### Evaluation Metrics

Our study leverages a variety of metrics to validate the experiments.

To evaluate the overall performance of BugRMSys , we use Acc@N and Mean Recriprocal Rank (*MRR*), which are widely used metrics for recommender systems (Ye et al. 2014; Zhou et al. 2012; Poerner et al. 2020).

**Acc@N:** Acc@N hit measures the retrieval precision over the top$_N$ recommended issues or reviews in the ranked list:

2 $$\begin{aligned} Acc@N = \frac{ \Sigma ^N_i(pair(i))}{LENGTH}, \end{aligned}$$

where *LENGTH* represents the length of tested ground truth; pair(i) means if the *i-th* issue *B* hit the *i-th* top$_N$ reviews relevant to issue *A*, if yes, *pair(i)* = 1, else 0. Overall, *Acc@N* is an approach used in previous research describing how often the issue in target *B* is among the top$_N$ Nearest Neighbours (by cosine) of a DistilBERT word space.

**MRR:** *MRR* is short for mean reciprocal rank and is a popular metric used to evaluate the efficiency of recommendation systems (Shani and Gunawardana 2011; Cames and II-Grants 2006; Mahmood et al. 2009). The equation of accuracy is described as follows:

3 $$\begin{aligned} MRR = \frac{1}{N}*\Sigma _{i=1}^{\left| N\right| }\frac{1}{rank_i}, \end{aligned}$$

where *N* is the length of ground truth; For the *i-th* issue (app A) in ground truth, rank$_i$ represents the position of recommended review which also relevant to issue in app B.

To better understand how we calculate Acc@N and MRR in this study, let’s provide examples for each.

Acc@N is calculated as follows: Let’s say we have five ground truth issues, and we’re looking at the top 3 (N$=$3) recommendations for each issue. For each issue, we check if the correct matching issue is within the top 3 recommendations. If it is, pair(i) is 1; if not, pair(i) is 0. For instance, for our 5 issues, if we get these results: [1, 0, 1, 1, 0], Acc@N would then be calculated as (1$+$0$+$1$+$1$+$0) / 5 $=$ 0.6. This means that 60% of the time, our model correctly includes the matching issue in the top 3 recommendations.

MRR (Mean Reciprocal Rank) is another metric we use. The MRR is calculated as follows: If we have three issues in the ground truth and their correct results are ranked 1st, 3rd, and 2nd in our model’s recommendations, the reciprocal ranks would be 1, 1/3, and 1/2, respectively. We sum these up and divide by the number of ground truths. In this case, MRR $=$ (1 $+$ 1/3 $+$ 1/2) / 3 $=$ 0.61, approximately.

### Implementation & Availability

We first developed two crawlers for automatically collecting bug reports and app reviews based on python packages: PyGithub^2^ and google play scraper^3^, respectively. We have implemented a prototype version of BugRMSys using Python (and associated frameworks) with a well-known, light-weight, transformer based, and contextual pre-trained model, DistilBERT, to extract vector representations for both bug reports and app reviews. The dataset and the replication package of BugRMSys are publicly available at:

https://github.com/Daniel4SE/BugRMSys

## Experimental Results

In this section, we conduct qualitative and quantitative analysis to evaluate BugRMSys . To this end, we evaluate the effectiveness of BugRMSys , we compare BugRMSys with state-of-the-art tools for bug recommendation, we study the characteristics of BugRMSys , and we explore the transferability of BugRMSys .

### [RQ-1]: Effectiveness of BugRMSys

*Setup for RQ-1:* In our approach, we didn’t assign explicit ranks to the ’ground truth’ reviews. The ranking emerged from the operation of our tool, BugRMSys , which processes each Firefox bug report and attempts to match reviews from the Brave app. For each bug report, BugRMSys generates a list of matched reviews, ordered based on its internal algorithm. We retained only the top three matched reviews for each bug report. The ‘actual rank’ of each review, therefore, is essentially determined by the position of the match in the list generated by BugRMSys . We manually verified these matched reviews to ensure their semantic similarity to the corresponding Brave bug report. Although no explicit ranks were assigned to the ‘ground truth’, this order of matches, as generated by BugRMSys , served as the de facto ranking.

To answer RQ1, due to the huge manual effort for assessing the similarity of app reviews with bug reports, we focus on a single same-category app pair (FireFox, Brave), where FireFox will be the input app and Brave the target app (i.e., we use bug reports from Firefox to find new bug reports in Brave by matching Brave user reviews). Note that in the running example (cf. Section 2), we had illustrated a bug case where Firefox was the target app and Brave was the input app. This is an additional argument that BugRMSys can explore the experience of any app and leverage it for any other same-category counterpart regardless of which app appears to have more historical data.

We start by building a ground truth dataset to assess the ability of BugRMSys to find relevant bug reports for the recommendation. To that end, the idea is to first try to find existing Brave bug reports that are similar to the ones of FireFox to build a set of pairs of similar bug reports $<BR_{Firefox},BR_{Brave}>$. Then, for each pair $<BR_{Firefox},BR_{Brave}>$, we rely on BugRMSys to identify Brave app reviews that match $BR_{Firefox}$. However, we only consider Brave app reviews which precede the creation time of the corresponding $BR_{Brave}$. Finally, we manually check if the identified Brave app reviews match the corresponding Brave bug report $BR_{Brave}$. This would indicate that BugRMSys would have been useful to automatically recommend the Firefox bug report as relevant to Brave. Especially, bug reports of Firefox should be created earlier than the corresponding bug reports’ creation of Brave if we want use Firefox’s information to explore potential bugs in Brave.

In practice, to build our set of similar bug reports, we randomly picked 3,000 bug reports from Firefox. By using a cosine similarity threshold of 0.91^4^, we were able to identify 81 bug report pairs $<BR_{Firefox},BR_{Brave}>$ where the Firefox and Brave bug reports are highly similar. Note that this ground truth construction may be too conservative: there are possibly other Brave bug reports that are also semantically similar to a given Firefox bug report.

Recall that the objective of BugRMSys is to match bug reports to app reviews. In this case, we assess whether, for each pair of the ground truth, we can match the Firefox bug report with app reviews that are relevant (based on human expertise^5^) to the associated Brave report. If so, we can conclude that BugRMSys would have been able to recommend the Brave bug report.

For our experiments, we applied BugRMSys on each of the 81 Firefox bug reports to match Brave app reviews. We retain only the top 3 matched reviews per bug report. Overall, among the 81 top$_1$ reviews recommended by BugRMSys , 21 could be confirmed as indeed semantically similar to the content in the Brave bug report associated in the ground truth pair.

We further confirm 32 and 38 (at Top 2 and Top 3 respectively) app review matches. Table 4 details our results by also providing the Accuracy and MRR scores. Overall, with Top 3 recommendations on a conservative ground truth, we reach almost 50% hit ratio. Note that, while BugRMSys matches Firefox bug reports with Brave app reviews, our effectiveness evaluation is to check whether the matched app reviews are semantically relevant for Brave bug reports. This is the practical and ultimate concern of our bug recommendation scheme.

**Table 4:** Results of Acc@N and MRR@N

81 relevant bug pairs out of 3K bugs from Firefox	@1	@2	@3
App Review Hits	21	32	38
Value of Acc@N(%)	25.93	39.51	46.91
Value of MRR@N(%)	25.93	26.50	35.19

“App review Hits" represents the number of times BugRMSys matches the relevant app reviews associated to the ground truth bug report of Brave: this is a proxy for estimating that BugRMSys would have been able to recommend the Firefox bug report as relevant to Brave

Table 7 presents an extract of our set of 81 similar bug reports pairs by focusing on three pairs, the corresponding app user reviews and the result of our manual analysis. The full results are detailed on our Github repository^6^.

The purpose of BugRMSys is to match bug reports with app reviews, leveraging information from one application to potentially discover relevant bugs in another. In this experiment, we simulated this process by using Firefox as the ”input” app and Brave as the ”target” app.

Here are the key steps we took:

- We built a ground truth set of bug report pairs that are semantically similar between Firefox and Brave. This was done by manually selecting 3,000 Firefox bug reports and identifying 81 pairs that had a high degree of similarity with Brave bug reports. The similarity was computed using a cosine similarity threshold of 0.91.
- For each of these bug report pairs, we used BugRMSys to identify Brave app reviews that matched the Firefox bug report. We made sure that the identified Brave app reviews were created before the corresponding Brave bug report.
- We manually checked if the identified Brave app reviews matched the corresponding Brave bug report. If they did, this indicated that BugRMSys would have been useful in automatically recommending the Firefox bug report as relevant to Brave.

In this way, the experimental setup mirrors the intended application of BugRMSys - using information from one app (in this case, Firefox) to identify potential bugs in another (Brave), based on the similarity of app reviews to bug reports. The results demonstrated that, even with a conservative approach to building the ground truth, BugRMSys was able to identify relevant matches in about 50% of cases.

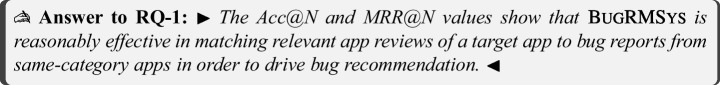



### [RQ-2]: Feasibility of BugRMSys

We conduct extensive execution of BugRMSys on data from 20 apps in 9 categories to recommend bugs. Due to space limitation, we report in Table 5 the statistics of bugs recommended by BugRMSys for the top$_{10}$ apps having the most recommended bugs. We note that, thanks to BugRMSys app-review driven approach, the collaborative bug finding allows to sift between a few hundreds to a few thousands bug reports from same-category apps in order to recommend^7^ only a few (1.63%) of bug reports as being relevant to the target apps.

**Table 5:** Ranked apps based on the number of potential bugs

Target	# of bug reports searched	# app reviews	# recommended
app	(from same category apps)	(for the target app)	bugs
Brave	10,000	10,000	208
Nextcloud	3,489	1,143	147
Wire	10,000	2,515	75
VLC	599	5,556	59
Firefox	10,000	10,000	52
Dolphin	2,437	2,498	44
Wordpress	4,139	2,714	44
PPSSPP	1,666	6,846	39
Mupen64Plus	2,437	1,401	39
Fbreader	726	3,914	33

Since each recommended bug is found by correlating information in its app reviews, we propose to estimate the potential time gain BugRMSys has brought by highlighting the buggy behaviour users complained about in unofficial channels. We compute the distribution of time elapsed since the app review creation date and the BugRMSys bug recommendation date. On average, specially for Firefox and Brave in Table 5, the app reviews were created 22.2 and 33 days before we submit the bug reports, respectively.

Given the labor-intensive nature of manual bug reproduction and submission, we have chosen to concentrate our investigation on four popular applications: Wire, Brave, Firefox, and Nextcloud. We’ve selected these application pairs - (Signal, Wire), (FireFox, Brave), and (Owncloud, Nextcloud) - to demonstrate the effectiveness of our approach. Our pipeline utilizes reports from the input applications to match reviews from the target application, implying that more matched reports suggest a greater likelihood of potential bugs in the target application.

As shown in Table 1, Signal has substantially more reports than Wire, suggesting it is more popular and likely has a more comprehensive set of bug reports. On the other hand, Firefox and Brave have comparable numbers of reports. As such, we selected Wire, Brave, and Firefox as the subjects of our investigation. Although Nextcloud and Owncloud have a similar number of bug reports and reviews, reproducing potential bugs in Owncloud would necessitate setting up a web server, increasing the complexity of the process. Therefore, we have chosen Nextcloud as our final subject.

**Table 6:** Previously unknown bugs detected with BugRMSys

Input app	Target app	# of bug reports (input apps)	# app reviews ( target app)	# recommended bugs	# of reproduction attempts	# successully reproduced bugs	# replied, confirmed or fixed bugs
Signal	Wire	10,000	2,515	75	12	2	2, [✔ 2]
Firefox	Brave	10,000	10,000	208	44	9	(1),2, [✔ 2]
Brave	Firefox	10,000	10,000	52	24	4	(1), 1
Owncloud	Nextcloud	3,489	1,143	147	12	5	(1), 1
Total	-	33,489	23,658	482	90	20	(3), 6, [✔ 4]

$^*$“(#)” means the number of reported bugs replied by developers but not confirmed or fixed by them

“[✔ #]” means the number of reported bugs confirmed and fixed by developers

Our methodology involved manually reproducing each recommended bug and submitting the successfully reproduced bugs to the respective application’s issue tracker. The numbers of recommended bugs, successfully reproduced bugs (selected based on their similarities, with the top-44 most similar ones chosen), and confirmed or fixed bugs are detailed in Table 6. You can find more details about the reported bugs on our repository^8^. We remind that we can recommend a bug report from app A (the first column in Table 6) as relevant to the target app B when BugRMSys matches the bug report of A with user app reviews from the target app B. We then use the “steps to reproduce” present in the bug report of A, as well as the information present in the app reviews of app B to reproduce the bug in the target app B. Finally, for each bug that has been successfully reproduced in the target app B, we submit the bug in the issue tracker of the app. As shown in the the last column of Table 6, six bugs have been already confirmed or fixed by the developers before this submission.

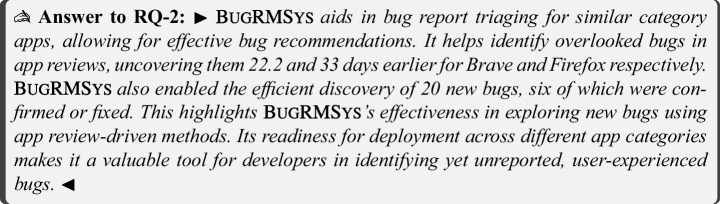



### [RQ-3]: BugRMSys vs Prior Works

Ideally, we should evaluate the performance of BugRMSys in comparison with prior works dealing with bug recommendations based on bug reports. There are two state of the art approaches, DeepMatcher (Haering et al. 2021) and Bugine (Tan and Li 2020), which are closely related.

DeepMatcher and BugRMSys both match app reviews with bug reports based on text embedding using pre-trained DistilBERT. Experimentally, we compare DeepMatcher against the BugRMSys by considering the ground truth data built for RQ-1 (cf. examples in Table 7): we propose to manually check whether the matched reviews with both approaches are relevant or not. While all reviews matched by BugRMSys are relevant to buggy behaviour, we observe that DeepMatcher only achieves a F1-score of 71% in filtering useful reviews. We postulate that BugRMSys performs better partly because it implements a focused collaborative bug finding approach where the matching is done on bug reports of same-category apps.

We also compare against Bugine, which also performs collaborative bug finding. We differ however as Bugine limits the matching to cases where apps have the UI/components (while we consider apps from teh same categories). We further consider app reviews to drive bug recommendation.

In the remainder, we further elaborate on the specific differences that prevent comparison between prior works and BugRMSys . These differences relate to three aspects: (1) Differences in input, output, and workflow; (2) Differences in automation level; (3) Performance in new bug finding.

**Table 7:** Extract of our Ground Truth Dataset, Corresponding relevant app reviews, and Manual check result

Existing Bug Reports (input, target)		TOP 3 RELEVANT REVIEWS in Brave *(creation time always prior to the corresponding Brave bug report)*	MANUAL
App: Firefox Data: 2020-08-21 Reports: Download does not work on a custom tab (Slack)	App: Brave Date: 2021-03-20 Report: Download [Status Bar] Improvement	Date: 2020-08-22 Review: Downloader is very bad....pls increase and more work on download manager	TRUE
		Date: 2020-08-10 Review: There is no download option in this could u pls update on this issue	TRUE
		Date: 2020-10-18 Review:...it does not allow to manually add download tasks	TRUE
App: Firefox Data: 2020-06-17 Reports: Report clickbait sites, Protect user privacy	App: Brave Date: 2020-06-22 Report: Warn users about insecure Facebook and Google privacy settings	Date: 2020-06-08 Review: Only browser that cheats about privacy. All claims about user privacy are bogus...	TRUE
		Date: 2020-11-01 Review: Extremely convoluted privacy practices. They advocate for privacy but allow certain creepy sites ...	FALSE
		Date: 2020-12-31 Review: Good privacy app. It doesn’t prevent websites from annoying redirections	FALSE
App: Firefox Data: 2020-08-28 Reports: Invalid URLs can be bookmarked and they crash the browser	App: Brave Date: 2020-11-06 Reports: Clicking URLs outside of Brave opens a blank browser window with no URL	Date: 2020-06-11 Review: "..., has come under fire for automatically redirecting URLs typed into the browser’s address bar ...	FALSE
		Date: 2020-10-20 Review: One of the best browsers Imo. Only wish I could set links on the brave homepage manually...	FALSE
		Date: 2020-10-13 Review: Its a good browser sometimes it reload all tabs when i open newly	FALSE

#### Differences in Input, Output, and Workflow

DeepMatcher employs user reviews for App B as input and recommends relevant bug reports for App B. The workflow is: App review $\rightarrow$ Problem report $\rightarrow$ Matched relevant bug reports. They only evaluate their tool on existing bugs instead of exploring new bugs. Furthermore, DeepMatcher does not leverage experience from other apps when investigating a target app. Their approach further suffers from the redundancy problem in app reviews.

Bugine employs issues in apps with same UI components as their database. They focus on building a automatic test generation from bug reproduction steps and run the test on target app with manual check. Bugine has been used to explore new bugs successfully. However, there is a great limitation in this approach: it only considers app issues with same-UI components into consideration, which can reduce the feasibility of learning from other apps.

#### Difference in Automation Level

Different from DeepMatcher, BugRMSys will not process a large number of bug reports: we focus on same-category apps to match relevant reviews of App B. After manual check, we have verified that when we feed a bug report into BugRMSys , the matched reviews are 100% related to some bugs. By building on same category apps (i.e., with similar functionality and usage steps) reproduction and localization of bugs is eased.

For Bugine, finding apps with same-UI components is a time-consuming task. In addition, using issues from same-UI apps makes it hard to transfer the learned expertience to other types of issues.

#### Performance in Bug Finding

The ability of DeepMatcher to find new bugs has not been evaluated. Bugine reported having found 34 new bugs in 5 evaluated apps. With BugRMSys , within a week, we were able to recommend, reproduce and identify 20 new bugs across 6 apps. 4 such bugs are already fixed by the app developers.

#### Reproducibility of Bugs and Performance

Of the identified bugs, we reproduced 20 out of 90, a success rate that speaks to the reliability of our method. It’s important to note, however, that reproducing bugs is a complex process with many variables, and we are continuously working on improving this aspect of our approach. We are particularly interested in exploring automated test case generation to enhance the efficiency of bug reproduction.

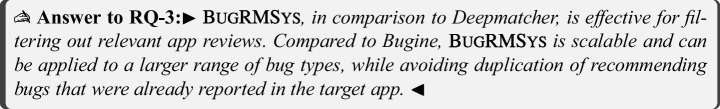



## Discussion

### Failures to Reproduce Recommended Bugs

As illustrated previously, some of the reproduction attempts on the bug reports recommended by BugRMSys . lead to failures There are various reasons that explain such failures without suggesting that the recommended bug is not relevant. Prior studies have already largely elaborated on this difficulty to reproduce bugs: In Han’s work (Han et al. 2019), an extensive classification of 8 categories of root causes for failed reproductions is provided: hardware dependency, operating system dependency, component dependency, unavailable source code, compilation error, installation error, missing step, and lack of symptom. Our failures causes span across these categories.

### Threats to Validity

Our design, implementation and evaluation of BugRMSys carries some threats to validity. First, when we are building the ground truth, we manually check whether the reviews are meaningful. Therefore, the ground truth may be biased by our own experience. Second, BugRMSys is not fully automated, i.e., manual effort is still needed when we reproduce from the recommended bug reports. Consequently, the success rate of reproduction could be dependent on the developing experience of individual developer. Risk of lacking independent assessors: As bug reproduction seems to heavily rely on users, there may be some bias in empirical evaluation.

## Related Work

**Collaborative Experience Sharing** Collaborative programming is common in the development of open source software. Consequently, similar bugs can emerge across different projects. Other researches attempted to leverage this fact to Recommend, Reproduce, and Repair inter-project bugs. For instance, the detection of duplicate bug reports has been studied in localizing fault of software (Sun et al. 2011; Wang et al. 2008). Specifically, Yang et al. (2016) combined the information retrieval technique and word embedding technique to process the detailed information of bug reports to recommend similar bugs. On the other hand, Tan and Li (2020) use three collaborative sources for bug finding: (1) bugs from the same programmer across different projects, (2) bugs from manually searching for bug reports in GitHub repositories, (3) bugs from a bug recommendation system. Based on these shared experiences, they explored the concept of collaborative bug finding on improving the teaching of software testing courses. In the experience-based collaborative learning of crowd-sourcing, Mao et al. (2017)’s experimental results show that the generated replicable test scripts from crowd-based testing can improve the coverage attainment for automated mobile testing.

**Recommendation of Bug Reports Based on App Reviews** The importance of app reviews in App vendors has been comprehensively demonstrated (Oh et al. 2013; Sefferman 2015). Leaving the app reviews not to be addressed is harmful to the experience of the users and rating of the app, and further lead to uninstallation of the app (Hassan et al. 2018). To maintain the evolution of the app, researchers started to leverage app reviews. For instance, Gao et al. (2019) developed a novel approach to automatically generate proper responses to the app reviews in Google Play. However, this approach mainly try to (1) soothe bad emotion of users, and (2) collect detailed user experience, but not to discover potential bugs in advance. Tan and Li (2020) designed an approach to find bugs for Android apps. Their pipeline is to retrieve bugs in other similar apps that may also exist in the current app. This work validates the feasibility of searching for bugs in other projects to identify new bugs mentioned by app reviews. Afterwards, Marlo et al. (Haering et al. 2021) try to match bug reports with related app reviews to discover bugs by filling the gap of different languages between app reviews written by non-technical users and bug reports proposed by professional developers.

## Conclusion and Future Work

In this paper, we introduce BugRMSys , a tool-supported approach for app reviews driven collaborative bug finding. Given a target app B, BugRMSys builds on the development experience of app A to identify bug reports in A that match app reviews of B. If such bug reports exist, they are considered as candidate for recommending bugs to the target app B. To that end, BugRMSys implements an embedding procedure to represent bug reports and app reviews text, and use cosine similarity to decide on matching similarity scores. Once bugs are recommend, we experimentally attempt reproduction to confirm the detection of new bugs in the target app. Our experimental results on free and open source apps in various categories show that BugRMSys is effective, scales to a variety of bug types, and does not yield too many irrelevant app review matches. Overall, with BugRMSys , we already successfully reproduced 20 new bugs in 6 apps across 3 categories. Several of these bugs have been acknowledge by the apps development communities and some have even already been fixed.

In future, we plan to address the question of automating the reproduction phase in order to scale the collaborative bug finding approach towards further increasing its practicality in real-world debugging scenarios.

## Figures and Tables

**Fig. 10:**
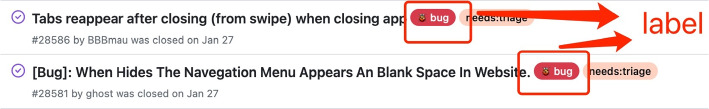
Labelling in GitHub system

## Data Availability

The datasets and code used in this work are available in the following link: https://zenodo.org/record/7520604#.Y71lnezML0o

## Appendix

### A.1 More Information about Dataset

Overall, we have 130 apps from the Wikipedia 9 classified in 15 categories, shown in Table 8. However, only few apps can provide both user reviews and bug reports in their GitHub system. We finally find out 10 apps that can provide enough reviews and bug reports (as shown in Table 5).

Choosing 3 apps from each of the two categories, and 2 apps from each of the remaining 7 categories is up to which one can provide both reviews and bug reports (even all listed apps are open source, but few of them provide google reviews or bug reports).

**Table 8:** All app categories

Domain Name	App Names	Number
Advertisement blocking	AdAway, Adblock Plus	2
Web browsers	Brave, Chromium, DuckDuckGo, Fennec F-Droid, Firefox for Anadorid, Firefox Focus, GNU IceCat, Tor Bowser	8
Office Suites	Collabora Online, Owncloud, Nextcloud	3
Privacy security focused	APG, Briar, Conversations, Element, I2P, IVPN, Jami, Lantern, Mullvad, OpenKeychain, Orbot, Psiphon, Protonmail, ProtonVPN, Signal, Surespot, Telegram, Tox, Tutanota, Wire	20
Communication	ConnectBot, CSipSimple, Jitsi, K-9 Mail, Linphone, Element, Sipdroid, WordPress, Zuilip	9
Emulators	Citra, Dolphin, Mupen64Plus, openMSX, PPSSPP, RetroArch, ScummVM, Termux, VICE	8
Games	2048, Angband, Battle for Wesnoth, Brogue, Dungeon Crawl Stone Soup, Fish Fillets NG, Freeciv, Frozen Bubble, GLtron, H-Craft Championship, HyperRgue, Mindustry, Minetest, OpenArena, OpenTTD, Pixel Dungeon, OpenTyrian, robotifindskitten, Simon Tatham’s Puzzle Collection, Ur-Quan Masters	20
General	Dasher, FetLife, Google IO, OpenLP, The White House	5
Health	COVID Alert, DP-3T, PEPP-PT, TraceTogether	4
Multimedia	AntennaPod, Butter Project, Kodi, NewPipe, Popcorn Time, Ringdroid, Rockbox, Tribier, Tux Paint, VLC, Wikimedia Commons	12
Navigation	Avare, MAPS.ME, Mozilla stumbler, OsmAnd	4
Reading	FBReader, iFixit, Kiwix, MuPDF, Wikipedia, Wikitionary, XOWA	7
Science and education	BOINC, Galaxy Zoo, GCompris, GNU Octave, micro:bit, PressureNET, SageMath, Sky Map, Stellarium, Sugar environment, AnkiDroid	13
Security	Bitwarden, Haven, Kali NetHunter, KeePassDroid, PasswdSafe, Prey	6
System and utilities	Barcode Scanner, F-Droid, Impress Remote, Intra, microG, Mycroft, TWRP, TalkBack, UserLand	9

### A.2 The Methodology for App Selection Across Categories

As shown in Table 8, we have 130 applications in 15 categories, while few applications are selected in our experiment, which is shown Table 3. This study proposes a novel methodology for the selection of applications from various categories, centered around three primary criteria.

- **GitHub Issue Accessibility:** The first criterion of selection is predicated on the applications providing an accessible issue feature within the GitHub system. This is significant because it gives users and developers an avenue to report problems, inquire about aspects of the application, and suggest new functionalities.
- **Ease of Use:** The second selection criterion is the ease of use of the applications. For an application to be considered ‘easy to use’, it should require minimal user effort to commence operation, i.e., either direct usage post-installation or usage following a straightforward registration process.
- **Sufficient Google Reviews:** The third criterion for application selection is the existence of a large number of Google reviews. This acts as a proxy for the general public’s interaction with and evaluation of the application, ensuring its widespread use and testing.

To illustrate these criteria, consider the following applications which failed to meet one or more of the aforementioned selection criteria:

- The ‘Tor Browser’, a candidate for the Web Browser category, was not selected due to the absence of an accessible issue feature within its GitHub repository.
- ‘Owncloud’, a contender for the Office Suites category, was not chosen as it failed to meet the ‘ease of use’ criterion, necessitating users to employ a web server to access the cloud functionality.
- ‘Talkback’, a potential selection for the System and Utilities category, was disqualified due to the lack of Google reviews, contravening the third selection criterion.

Through the application of these three criteria, a robust selection methodology can be developed for applications across various categories. This approach ensures the selection of apps that are user-friendly, have active developer interaction and have been sufficiently reviewed by the wider user community.

### A.3 Ground Truth Construction

The establishment of the ground truth for evaluating the effectiveness of BugRMSys involved a rigorous process outlined as follows:

1. **Selection of Bug Reports:** We initiated our study by randomly selecting a sample of 3,000 bug reports from Firefox GitHub repository, avoiding potential bias that may occur using a manual selection process.
2. **Identification of Similar Bug Report Pairs:** Utilizing an empirically selected cosine similarity threshold of 0.91, we identified 81 pairs of highly similar bug reports between Firefox and Brave, denoted as BR$_{Firefox}$, BR$_{Brave}$, ensuring a high-quality and conservative dataset based on empirical validation.
3. **Matching with Preceding App Reviews:** For each bug report pair, BugRMSys was tasked with matching Firefox bug reports to pre-existing Brave app reviews, predating the Brave bug report creation. This step underscored the temporal relevance of the identified matches for practical bug identification.
4. **Manual Verification:** The top three matched reviews for each bug report were manually verified by three software engineers against the corresponding Brave bug report for semantic similarity. This manual verification ensured the relevance and feasibility of the matches identified by BugRMSys .

**Significance of the Ground Truth:**

The ground truth established through this process serves as a critical empirical foundation for evaluating BugRMSys ’s capability in leveraging user reviews and bug reports from a similar app to identify and recommend relevant bug reports. The methodology emphasizes:

- *Empirical Basis for Tool Evaluation:* Offering a practical scenario where BugRMSys can utilize historical data and user feedback for proactive bug identification in another app.
- *Relevance and Timeliness:* Highlighting the tool’s potential to leverage insights from user reviews and similar apps’ bug reports to aid in timely bug identification and resolution.
- *Quality over Quantity:* The high similarity threshold and manual verification ensure that the dataset represents high-quality, semantically similar matches, crucial for assessing BugRMSys’s effectiveness beyond mere superficial matches..

### A.4 Bug Report Identification in Issue Reports

The bug reports and user reviews are selected based on the cosine similarity with a given text input. The code computes embeddings (numerical representations) for the input text and for a set of issue reports and user reviews. It then measures the cosine similarity between the embedding of the input text and the embeddings of each issue or review. Cosine similarity is a metric used to measure how similar two vectors are. In this case, it is used to find the similarity between the embeddings of the input text and the issues or reviews.

This selection process aims to find the most relevant bug reports and user reviews based on the content of the input text by computing and comparing their cosine similarities.

As shown in Fig. 10, we differentiate bugs and the other issues by label filtering. Label Filtering: GitHub and other issue tracking systems allow us to label issues. For example, I choose a issue labelled as a ‘bug’, ‘enhancement’, ‘documentation’, etc. Filtering issues by the ‘bug’ label is an efficient way to separate bugs from other issues.

### A.5 Data Preprocessing

In the initial phase of our methodology, data preprocessing is performed to enhance the quality and relevance of our data inputs, particularly in the context of app reviews and bug reports. This involves several steps:

1. **Normalization:** All tokens are converted to lowercase to avoid differentiation based on letter casing.
2. **Noise Reduction:** Elements such as punctuation, stop words, digits, and emoticons are removed to streamline the content. This ensures the focus remains on key text data.
3. **Spelling Correction and Word Repetition:** Given that app reviews and bug reports may contain spelling mistakes and repetitive words, we utilize rule-based methods to identify and rectify these issues.
4. **Length-based Filtering:** Reviews are filtered based on their length. Those that are either too short (fewer than 10 words) or too long (based on outlier thresholds) are excluded. Short reviews often lack substantive content, while excessively long ones could introduce noise into the data.

For bug reports, our approach primarily focuses on their titles, as they typically encapsulate the essential content of the report. Other sections of the bug reports, such as the description and reproduction steps, often contain significant noise or templated data that may not contribute meaningfully to the bug matching process.

This comprehensive preprocessing stage refines our input data by reducing noise and increasing the relevance of the text content for subsequent processing stages, ensuring the effectiveness of our approach.

## Footnotes

1 https://en.wikipedia.org/wiki/List_of_free_and_open-source_Android_applications

2 https://pypi.org/project/PyGithub/

3 https://pypi.org/project/google-play-scraper/

4 We decided on 0.91 based on an **empirical validation**: the higher the similarity threshold is, the less pairs will be matched, and at the same time matched reviews are closer to bug reports. We decide on a high threshold to maximize high-quality results.

5 This is the core challenge of BugRMSys: to find semantic similarity between a bug report and an app review

6 https://github.com/BugRMSys/BugRMSys/blob/main/RQ1UserCase_firefox2brave.csv

7 We set a high similarity threshold at 0.9. This value can be fine-tuned following the practitioner’s objectives.

8 https://github.com/Daniel4SE/BugRMSys/blob/main/new_bugs.md
